# Natural Products and Skin Diseases

**DOI:** 10.3390/molecules26154489

**Published:** 2021-07-25

**Authors:** Juraj Majtan, Marcela Bucekova, Milos Jesenak

**Affiliations:** 1 Institute of Molecular Biology, Slovak Academy of Sciences, Dubravska Cesta 21, 845 51 Bratislava, Slovakia; marcela.bucekova@savba.sk; 2 Department of Microbiology, Faculty of Medicine, Limbova 12, 833 03 Bratislava, Slovakia; 3 Department of Paediatrics, Jessenius Faculty of Medicine, Comenius University in Bratislava, University of Teaching Hospital in Martin, Kollarova 2, 036 59 Martin, Slovakia; jesenak@gmail.com; 4 Department of Clinical Immunology and Allergology, Jessenius Faculty of Medicine, Comenius University in Bratislava, University of Teaching Hospital in Martin, Kollarova 2, 036 59 Martin, Slovakia

The skin is the largest multifunctional organ in the human body, serving as an excellent barrier against chemical and biological hazards. Skin diseases present a major health concern worldwide, and are caused by both intrinsic and extrinsic factors with no distinctions of either age or ethnicity [1]. Currently, there are a number of therapies used for skin disorders; however, they pose several limitations, such as adverse effects or limited penetration.

Nowadays, there is growing interest in identifying novel, low-cost, highly effective and safe molecules that may be used in the treatment of skin disorders, especially chronic inflammatory diseases of the skin, such as atopic dermatitis (AD) and psoriasis. One of the potential sources of biologically active agents are the natural products of plants, insects and animals. Several natural molecules, encapsulated in lipid nanosystems, have been considered in the treatment of some skin pathologies or diseases [2].

The aim of the present special issue, entitled ‘Natural Products and Skin Diseases’ is to characterise new approaches in the treatment of skin disorders by collecting together papers that focus on such topics as skin penetration and photoageing, AD, wound healing, oxidative stress, melanogenesis, skin damage and cosmetics.

The rigorous peer review process resulted in the acceptance of 13 manuscripts out of 25 submitted by scientists from different countries. The high number of submissions clearly indicates that it is an interesting area of research attracting many research groups.

An important part of the special issue is related to skincare products, including cosmetics, their efficacy and safety. Despite the requirements for safety, mercury (Hg) and its compounds still appear in skincare products, including those of high quality. A study by Polish researchers [3] focused on the evaluation of Hg content in face and body skincare products. Interestingly, face preparations contained a significant higher amount of Hg compared to body preparations. No differences in the content of the element were found between natural and conventional preparations. Continuous determination of Hg, as well as other elements such as lead, cadmium or chromium is necessary to secure the safe use of skincare products.

Excessive use of cleansing cosmetics may result in problems with atopic skin due to their ability to disturb the hydrophilic barrier and thus cause skin irritations. Bujak and co-workers [4] investigated the effect of ectoine, a rare amino acid produced by extremophiles, on the irritation potential of anionic surfactants. Supplementation of ectoine in surfactant solutions showed a significant reduction in their cytotoxicity and decreased irritation potential. It is also able to protect cells against oxidative and osmotic stress, which can stimulate proliferation and increase metabolic activity of those cells. These results suggest that the addition of ectoine to anionic surfactant solutions improves the safety of cleansing cosmetics.

Numbers of cosmetic preparations and skincare products contain hyaluronic acid, an important small molecule that fills the spaces between collagen and elastin fibres and helps the maintenance of skin moisture. The content of hyaluronic acid is regulated by the action of the enzyme hyaluronidase, and suppressing enzyme activity results in reduced wrinkle formation. Mohamed et al. analysed various parts of the plant *Ravenala madagascariensis* for their inhibitory activity against hyaluronidase [5]. The docking study showed that narcissin, rutin and quercetin 3-*O*-glucoside all affect hyaluronidase activity. The flavonoid content of *R. madagascariensis* presents promising hyaluronidase inhibitors that could be used in natural cosmetology preparations for skincare.

An attractive area of research concerns identified and/or synthesised melanin synthesis inhibitors with no side effects or cytotoxicity. Kim et al., identified neobavaisoflavone in the medicinal plant *Pueraria lobata*, which possesses depigmentation activity through the inhibition of melanogenesis [6]. It was showed that neobavaisoflavone induces phosphorylation of GSK and ERK signals and subsequently decreases melanin synthesis. These results suggest that neobavaisoflavone may be a useful depigmentation compound and a novel alternative in the medical and cosmetics industries.

Nowadays, there is growing interest in the search for novel, effective and safe dermatological preparations containing active ingredients with multiple effects. Plant extracts can exhibit multiple effects, such as antibacterial, antioxidant, anti-inflammatory, antioxidant and anti-ageing activities due to the abundance of secondary metabolites. Therefore, plant extracts are interesting sources of biologically active compounds that may be used as components of cosmetic and dermatological preparations. One ethanolic plant extract, from the well-known medicinal plant *Epilobium angustifolium*, or fireweed, exhibits anti-collagenase, anti-elastase and anti-inflammatory effects [7]. In addition, active ingredients of the plant extract (phenolic acids) were able to penetrate through human skin and be accumulated in it. In the case of plant substances such as phenolic acids, their greater accumulation in the skin is preferred, where they exhibit anti-ageing effects. Penetration and subsequent accumulation of active compounds in the skin is an attractive research area that opens up new avenues in the treatment of skin disorders. Therefore, the authors of the above-mentioned study also investigated the in vitro human skin penetration and antibacterial properties of the ethanol-water extract of medicinal plant *E. angustifolium* [8]. The antibacterial activity of the extract was superior against bacteria of the genera *Serratia* and *Bacilllus* compare to those of the genera *Enterococcus, Streptococcus* and *Pseudomonas*. Obtained results also showed that isolated compounds, namely gallic acid, chlorogenic acid and 3,4-dihydroxybenzoic acid, possessed high antioxidant activities and exhibited the highest skin penetration potential. The authors concluded that fireweed extract could be used as an ingredient in cosmetics and pharmaceutics, providing multiple beneficial biological actions.

Diverse biological properties of medicinal plants are also employed in the wound healing process. Many extracts of medicinal plants including *Marantodes pumilum* have been tested for their wound healing activity. This was tested in vivo for its ability to promote wound healing in a rat model [9]. A histological analysis revealed better re-epithelialisation, enhanced fibronectin content and fibroblast cells, as well as higher fibre transformation from collagen-III to collagen-I accompanied by an abatement of inflammatory cells in the granulation tissues. Furthermore, antioxidant effects of the extract may have enhanced wound healing in the rat model.

Three papers published in special issue focused on the prevention of skin damage and photoageing. In particular, solar exposure of the skin accounts for up to 90% of skin damage. Jin et al., conducted research concentrating on the anti-photoageing effect *of Acer tataricum* subsp. Ginnala in ultraviolet B (UVB)-irradiated human fibroblasts [10]. The water extract of *A. tataricum* downregulated the expression of UVB-increased pro-inflammatory cytokines and also stimulated the TGFβ/Smad pathway that plays a critical role in promoting collagen synthesis suggesting that *A. tataricum* extract is a functional material with anti-photoageing properties. It is well documented that UVB induces cytotoxicity and the production of metalloproteinases (MMPs) and reactive oxygen species (ROS). Likewise, medicinal plant extracts such as exopolysaccharides produced by lactic acid bacteria, namely *Lactobacillus plantarum* HY7714, effectively counteract UVB-induced damage and increase the moisture retention in human fibroblasts [11]. These observations, investigated by Lee et al., suggest that exopolysaccharides, particularly from *L. plantarum*, are anti-ageing molecules and could serve as functional substances in skin–gut axis communication. The third paper focusing on skin protection was published by Wang et al. [12]. Diphlorethohydroxycarmalol, an algal polyphenol isolated from the edible brown seaweed *Ishige okamurae*, showed protective effects against particulate matter-induced skin damage in human fibroblasts. The authors reported that the compound reduced intracellular ROS generation in fibroblasts and also induced collagen synthesis. It could be used as an ingredient in the pharmaceutical and cosmeceutical industries.

Prolonged oxidative stress often induces an imbalance between the production and elimination of ROS that may result in chronic inflammation and cause acute and chronic skin diseases. High glucose content is considered as a stress-induced pro-inflammatory factor. In this context, Do and co-workers [13] characterised the in vitro anti-oxidative and anti-inflammatory effects of ethanolic extract of camu-camu fruit (*Myrciaria dubia*). The fruit extract modulated the mitogen-activated protein kinase MAPK/activator protein-1 (AP-1), nuclear factor kappa-light-chain-enhancer of activated B cells (NF-κB), and nuclear factor of activated T cells (NFAT signaling) pathways related to inflammation, by downregulating the expression of pro-inflammatory cytokines and chemokines. Furthermore, camu-camu fruit treatment activated the expression of nuclear factor E2-related factor 2 (Nrf2) to protect keratinocytes against high-glucose-induced oxidative stress. These results indicate that camu-camu fruit is a promising material for preventing oxidative stress and skin inflammation induced by high glucose levels.

Of particular interest in the present special issue is the study of AD, a common inflammatory skin disease, the prevalence of which has increased over the past decades. Two research articles focused on characterisation of anti-atopic effects and mechanisms of action of deacetylasperulosidic acid, a major biologically active ingredient of the medicinal plant noni (*Morinda citrifolia*) [14] and piperine, a major alkaloid of long pepper *Piper longum*) and black pepper (*Piper nigrum*) [15]. Deacetylasperulosidic acid inhibits the secretion of AD-related cytokines and chemokines and increases the expression of proteins involved in skin barrier functions. These results confirmed that deacetylasperulosidic acid could relieve AD by controlling immune balance and recovering skin barrier function. Similarly, piperine inhibits the expression of pro-inflammatory cytokines and suppresses Type 2 helper-mediated immune responses, including the STAT6/GATA3/IL-4 signalling pathway. Topical treatment with piperine reduced AD symptoms in an AD-like mouse model.

In conclusion, this special issue was dedicated to natural products, their active ingredients and their role in the treatment of skin diseases. We believe that this special issue will stimulate further research in this interesting area.
